# Three new wood-inhabiting fungal species of *Phanerochaetaceae* (*Polyporales*, *Basidiomycota*) from Southwest China

**DOI:** 10.3897/mycokeys.139.199706

**Published:** 2026-08-26

**Authors:** Qi Li, Shunhong Wang, Jian Feng, Chengwei Shu, Changlin Zhao

**Affiliations:** 1 The Key Laboratory of Forest Resources Conservation and Utilization in the Southwest Mountains of China Ministry of Education, Yunnan Provincial Key Laboratory for Conservation and Utilization of In-forest Resource, Southwest Forestry University, Kunming 650224, China Department Microbial Drugs (MWIS), Helmholtz-Centre for Infection Research Braunschweig Germany; 2 College of Forestry, Southwest Forestry University, Kunming 650224, China College of Forestry, Southwest Forestry University Kunming China; 3 Forestry and Grassland Bureau of Jinggu Dai and Yi Autonomous County, Puer 665000, Yunnan, China The Key Laboratory of Forest Resources Conservation and Utilization in the Southwest Mountains of China Ministry of Education, Yunnan Provincial Key Laboratory for Conservation and Utilization of In-forest Resource, Southwest Forestry University Kunming China; 4 Department Microbial Drugs (MWIS), Helmholtz-Centre for Infection Research, 38124 Braunschweig, Germany Forestry and Grassland Bureau of Jinggu Dai and Yi Autonomous County Puer China

**Keywords:** Biodiversity, corticioid fungi, new taxon, *
Polyporales
*, taxonomy

## Abstract

Three new wood-inhabiting corticioid fungal species from Southwest China, *Phanerochaete albomarginata*, *P. nigella*, and *Rhizochaete membranacea*, are proposed based on a combination of morphological features and molecular evidence. *Phanerochaete albomarginata* is characterized by membranaceous basidiomata, a monomitic hyphal system, generative hyphae with simple septa, tapering cystidia, and ellipsoid basidiospores. *Phanerochaete nigella* is characterized by its coriaceous basidiomata with a tuberculate hymenial surface, tubular cystidia, and ellipsoid basidiospores measuring 4.7–5.7 × 2.5–3.2 µm. *Rhizochaete membranacea* is characterized by membranaceous basidiomata, a monomitic hyphal system, generative hyphae encrusted with crystals, and ellipsoid basidiospores (4–5.5 × 2.7–3.1 µm). Phylogenetic analyses using Maximum likelihood (ML) and Bayesian inference (BI) were conducted based on ITS + nLSU markers. The resulting ITS + nLSU phylogram revealed that *P. albomarginata* formed a monophyletic lineage closely related to *P. taiwaniana* and *P. yingjiangensis*, while *P. nigella* formed a sister lineage to *P. albida*. Additionally, the phylogenetic tree of *Rhizochaete* inferred from the ITS + nLSU sequences showed that the new species formed a distinct branch and was sister to *R. yunnanensis*. This discovery enriches the known diversity of *Phanerochaetaceae* in subtropical Asia and further clarifies the phylogenetic relationships within the family.

## Introduction

In ecosystems, wood-inhabiting fungi occur on living trees, dead standing trees, fallen decorticated trunks, fallen trunks and branches, stumps, and even manufactured wood products (Spirin et al. 2017; Larsson et al. 2025). These fungi secrete various enzymes that efficiently break down cellulose, hemicellulose, and lignin into simple inorganic substances, thus playing a key role as decomposers in forest ecosystems (Larsson 2007; Dai et al. 2021). These fungi are functionally categorized as white-rot or brown-rot taxa based on decay patterns (Bucher et al. 2004).

The family *Phanerochaetaceae* Jülich is a major group of wood-inhabiting fungi (*Basidiomycota*) characterized by relatively simple basidiomata and fewer diagnostic morphological features than those of polypores and mushrooms (Floudas and Hibbett 2015). *Phanerochaetaceae* belongs to the order *Polyporales (Basidiomycota)* and is typified by *Phanerochaete* P. Karst. Twenty-nine genera have been placed in *Phanerochaetaceae*: *Alboefibula* C.C. Chen & Sheng H. Wu, *Bjerkandera* P. Karst., *Callosus* C.L. Zhao, *Cremeoderma* Sheng H. Wu & C.C. Chen, *Crepatura* C.L. Zhao, *Cystidichaete* X.C. Zhang & C.L. Zhao, *Donkia* Pilát, *Donkiella* J.H. Dong & C.L. Zhao, *Efibulella* Zmitr., *Gelatinofungus* Sheng H. Wu et al., *Geliporus* Yuan Yuan et al., *Hapalopilus* P. Karst., *Hyphodermella* J. Erikss. & Ryvarden, *Odontoefibula* C.C. Chen & Sheng H. Wu, *Oxychaete* Miettinen, *Paradonkia* Y. Xu & C.L. Zhao, *Neodonkiella* Y. Xu & C.L. Zhao, *Phaeophlebiopsis* Floudas & Hibbett, *Phanerina* Miettinen, *Phanerochaete* P. Karst., *Phlebiopsis* Jülich, *Pirex* Hjortstam & Ryvarden, *Porostereum* Pilát, *Quasiphlebia* C.C. Chen & Sheng H. Wu, *Rhizochaete* Gresl., Nakasone & Rajchenb., *Riopa* D.A. Reid, *Roseograndinia* Hjortstam & Ryvarden, *Stereophlebia* Zmitr., and *Terana* Adans, according to recent studies (Dong et al. 2024; Larsson et al. 2025).

*Phanerochaete* is a large, morphologically diverse genus within *Phanerochaetaceae*, typified by *P. alnea* (Fr.) P. Karst. and widely distributed from boreal to tropical forests worldwide (Spirin et al. 2017; Luo et al. 2024). It is characterized by resupinate, membranaceous basidiomata, a smooth or tuberculate hymenial surface, a monomitic hyphal system, generative hyphae mostly with simple septa, the presence of smooth or encrusted cystidia, and thin-walled, non-amyloid, and acyanophilous basidiospores, and causes white rot (Wu 2000; Floudas and Hibbett 2015). The diversity and taxonomy of *Phanerochaete* s.l. in China have been studied for 30 years (Wu 1990; Ghobad-Nejhad et al. 2015; Zhang et al. 2023). Early studies of fungi were largely based on morphology. Taxonomists used the membranaceous nature of the basidiomata, the monomitic hyphal system, the presence of clamp connections (simple, double, or multiple clamps per septum) or simple septa, and the presence of cystidia as characters for delimiting the genus. The simplicity of the morphological features characterizes *Phanerochaete*, and the presence of species with basidiomata that meet only some of these criteria renders the limits of the genus uncertain (Floudas and Hibbett 2015). With the advent of molecular biology, the taxonomic status of the genus *Phanerochaete* has become progressively resolved. Recent research, supported by morphology and phylogenetic analyses, has clarified the taxonomic status of many new taxa of *Phanerochaete* s.s. (Floudas and Hibbett 2015; Wang and Zhao 2021). Based on Index Fungorum (www.indexfungorum.org; accessed 19 August 2026), 228 species epithets of *Phanerochaete* are registered.

The genus *Rhizochaete* is a small, distinctive genus of wood-decaying fungi introduced by Greslebin et al. (2004) that produces hyphal cords and has a worldwide distribution (Gu and Zhao 2021). The genus, typified by *R. brunnea*, is characterized by resupinate to effused, loosely adnate, fragile basidiomata with a smooth to tuberculate hymenial surface (yellow, orange, brown, olivaceous, or violaceous, turning red to violet in KOH); a fimbriate to fibrillose margin, often with hyphal cords; a monomitic hyphal system with simple septa or clamps; cystidia usually present; clavate to subcylindrical, 4-sterigmate basidia; cylindrical to ellipsoid, smooth, thin- to slightly thick-walled, acyanophilous, Melzer-negative basidiospores; and growth on angiosperm and gymnosperm wood, causing white rot (Nakasone et al. 2017). Since *Rhizochaete* was erected, the number of newly named species has increased continuously. Several studies have contributed to knowledge of the genus *Rhizochaete*: Bianchinotti et al. (2005) found that three species possess perforate dolipore caps; Nakasone et al. (2017) described a new species from Belize and transferred three additional species based on morphological and molecular data; Gu and Zhao (2021) added two new species; Zhang et al. (2023) described *R. variegata* from Southwest China; and Li et al. (2023) recognized four new species (*R. nakasoneae*, *R. subradicata*, *R. terrestris*, and *R. yunnanensis*) mainly based on molecular analyses. In the phylogenetic tree of *Phanerochaetaceae* constructed by Larsson et al. (2025) based on ITS and LSU sequences, the *Rhizochaete* clade received moderate support, confirming the genus as an independent lineage within *Phanerochaetaceae*, and *Phanerochaete galactites* was transferred to the genus *Rhizochaete*. Based on Index Fungorum (www.indexfungorum.org; accessed 19 August 2026), 28 records of *Rhizochaete* are registered.

During investigations of the diversity of wood-inhabiting fungi, three new taxa from the genera *Phanerochaete* and *Rhizochaete* were collected from Yunnan Province, China. To clarify their phylogenetic placement and taxonomic relationships, morphological examinations and phylogenetic analyses of both genera were conducted using ITS + nLSU sequences.

## Materials and methods

### Sample collection and herbarium specimen preparation

Fresh specimens of wood-inhabiting fungi on angiosperm branches were collected from broad-leaved forests in Puer and Zhaotong, Yunnan Province, China. The samples were photographed *in situ*, metadata were recorded (Rathnayaka et al. 2025), and fresh macroscopic details were documented according to the guidelines provided by Dong et al. (2024) and Yang et al. (2025). All photographs taken in the field were focus-stacked using Helicon Focus software. Specimens were dried in an electric food dehydrator at 40 °C. Dried specimens were sealed in envelopes and zip-lock plastic bags and labeled with voucher numbers (Dong et al. 2025). The specimens are currently deposited in the herbarium of Southwest Forestry University (SWFC), Kunming, Yunnan Province, P.R. China.

### Morphological study

Macromorphological descriptions relied on field notes and photographs taken both in the field and in the laboratory. Color terminology followed Petersen (1996). Using a light microscope at 1000× magnification (with a 100× oil-immersion objective and a 10× ocular lens), micromorphological data were obtained from dried specimens (Dong et al. 2024). Sections were mounted in 5% KOH and 2% Phloxine B (C_20_H_2_Br_4_Cl_4_Na_2_O_5_) for microscopic observation, and micromorphological structures were additionally examined using Cotton Blue (CB) and Melzer’s reagent (IKI) to determine whether the measurements (e.g., spore sizes) changed in CB, IKI, and KOH. To show variation in spore sizes, 5% of the measurements were excluded from each end of the range and are shown in parentheses. At least 30 basidiospores from each specimen were measured. Stalks were excluded from basidia measurements, and the hilar appendage was excluded from basidiospore measurements (Yuan and Zhao 2024). The following abbreviations are used: KOH = 5% aqueous potassium hydroxide solution, CB = Cotton Blue, CB– = acyanophilous, IKI = Melzer’s reagent, IKI– = neither amyloid nor dextrinoid, *L* = mean spore length (arithmetic average for all spores), *W* = mean spore width (arithmetic average for all spores), *Qm* = average *Q* of basidiospores measured ± standard deviation, and *n* = *a*/*b* (number of spores [*a*] measured from a given number [*b*] of specimens). The MycoBank numbers for the new species were registered in MycoBank (http://www.mycobank.org).

### DNA extraction, PCR amplification, and sequencing

Fungal specimens were processed for genomic DNA extraction with the HiPure Fungal DNA Mini Kit II (Meiji Biotechnologies Co. Ltd., Guangzhou), which employs a rapid cetyltrimethylammonium bromide (CTAB)-based method. The extracted DNA was maintained at –20 °C for long-term storage. Two molecular markers were investigated: the internal transcribed spacer (ITS) region and the nuclear large subunit ribosomal RNA (nLSU) gene. The ITS region was amplified with the primer pair ITS5/ITS4 (White et al. 1990), and the nLSU region with the primer pair LR0R/LR7 (Vilgalys and Hester 1990). The PCR cycling procedure for ITS was as follows: an initial denaturation at 95 °C for 3 min, followed by 35 cycles at 94 °C for 40 s, 58 °C for 45 s, and 72 °C for 1 min, and a final extension at 72 °C for 10 min. The PCR procedure for nLSU was as follows: an initial denaturation at 94 °C for 1 min, followed by 35 cycles at 94 °C for 30 s, 48 °C for 1 min, and 72 °C for 1.5 min, and a final extension at 72 °C for 10 min. The PCR products were purified and directly sequenced at Kunming Tsingke Biological Technology Co. Ltd. (Yunnan, P.R. China). All newly generated sequences were deposited in NCBI GenBank (https://www.ncbi.nlm.nih.gov/genbank/) (Table 1).

**Table 1.** Names, voucher numbers, localities, references, and corresponding GenBank accession numbers of taxa used in this study. New species are shown in bold; * refers to type material (holotype), and—indicates missing data.**Qm**

**Species name**	**Specimen no**.	**GenBank accession no**.		**Country**	**References**
		**ITS**	**nLSU**		
*Alboefibula bambusicola*	Chen 2304*	MZ636926	MZ637091	China	Chen et al. (2021)
*Alboefibula bambusicola*	Wu 1209-26	MZ636927	MZ637092	China	Chen et al. (2021)
*Alboefibula gracilis*	Wu 0606-83	MZ636928	MZ637093	China	Chen et al. (2021)
*Alboefibula gracilis*	Wu 1809-106*	MZ636929	MZ637094	China	Chen et al. (2021)
*Bjerkandera adusta*	HHB-12826-Sp	KP134983	KP135198	USA	Floudas and Hibbett (2015)
*Bjerkandera centroamericana*	L-13104-sp	KY948791	KY948855	Costa Rica	Justo et al. (2017)
*Cremeoderma unicum*	Wu 1707-94	MZ636939	MZ637102	China	Chen et al. (2021)
*Cremeoderma unicum*	Wu 1707-100	MZ636938	MZ637101	China	Chen et al. (2021)
*Crepatura ellipsospora*	CLZhao 1265*	MK343692	MK343696	China	Ma and Zhao (2019)
*Crepatura ellipsospora*	CLZhao 1260	MK343693	MK343697	China	Ma and Zhao (2019)
*Cystidichaete alba*	CLZhao 39422	PX092380	PX092378	China	Zhang et al. (2026)
*Cystidichaete alba*	CLZhao 39667*	PX092381	PX092379	China	Zhang et al. (2026)
*Donkia pulcherrima*	GC 1707-11	LC378994	LC379152	China	Chen et al. (2018)
*Donkia pulcherrima*	Gothenburg-2022	KX752591	KX752591	Austria	Miettinen et al. (2016)
*Donkiella yunnanensis*	CLZhao 3931*	OR094482	OR461467	China	Dong et al. (2024)
*Donkiella yunnanensis*	CLZhao 18292	OR094483	OR461468	China	Dong et al. (2024)
*Efibulella deflectens*	FCUG 1568	AF141619	AF141619	Sweden	Parmasto and Hallenberg (2000)
*Gelatinofungus brunneus*	GC 1703-31*	LC387339	LC387344	China	Chen et al. (2018)
*Gelatinofungus brunneus*	Wu 1207-162	MZ636978	MZ637139	China	Chen et al. (2021)
*Geliporus exilisporus*	Dai 2172	KU598211	KU598216	China	Zhang et al. (2026)
*Geliporus exilisporus*	GC 1702-15	LC378995	LC379153	China	Chen et al. (2018)
*Hapalopilus eupatorii*	Dammrich 10744	KX752620	KX752620	Germany	Miettinen et al. (2016)
*Hapalopilus percoctus*	Miettinen 2008	KX752597	KX752597	Botswana	Miettinen et al. (2016)
*Hapalopilus nidulans*	JV 0206/2	KX752623	KX752623	Sweden	Miettinen et al. (2016)
*Hapalopilus rutilans*	FP-102473-Sp	MZ636981	MZ637142	USA	Chen et al. (2021)
*Hyphodermella corrugata*	MA: Fungi 24238	FN600378	JN939586	Portugal	Telleria et al. (2010)
*Hyphodermella laevigata*	He 5427*	ON964013	ON963996	China	Li et al. (2023)
*Hyphodermella laevigata*	He 5430	ON964014	ON963997	China	Li et al. (2023)
*Hyphodermella rosae*	GC 1604-113	MZ636986	MZ637147	China	Chen et al. (2021)
*Hyphodermella tropica*	He 3993*	ON964011	ON963994	China	Li et al. (2023)
*Hyphodermella tropica*	He 4004	ON964012	ON963995	China	Li et al. (2023)
*Irpex latemarginatus*	FP-55521-T	KP135024	KP135202	USA	Floudas and Hibbett (2015)
*Neodonkiella yinjiangensis*	CLZhao 30585*	PQ527892	PQ527889	China	Xu et al. (2025)
*Odontoefibula orientalis*	Wu 0910-57*	LC363490	LC363495	China	Chen et al. (2018)
*Odontoefibula orientalis*	GC 1703-76	LC379004	LC379156	China	Chen et al. (2018)
*Oxychaete cervinogilva*	GC 1501-16	MZ422783	MZ637173	China	Chen et al. (2021)
*Oxychaete cervinogilva*	Dmitry Schigel 5216	KX752596	KX752596	Australia	Chen et al. (2021)
*Paradonkia farinacea*	CLZhao 27184*	PQ527890	PQ527887	China	Xu et al. (2025)
*Paradonkia farinacea*	CLZhao 27221	PQ527891	PQ527888	China	Xu et al. (2025)
*Phaeophlebiopsis caribbeana*	HHB-6990	KP135415	KP135243	USA	Floudas and Hibbett (2015)
*Phaeophlebiopsis peniophoroides*	FP-150577	KP135417	KP135273	USA	Floudas and Hibbett (2015)
*Phanerina mellea*	Wu 1010-34	MZ422784	MZ637176	China	Chen et al. (2021)
*Phanerochaete aculeata*	Wu 1809-278	MZ422786	MZ637178	China	Chen et al. (2021)
*Phanerochaete aculeata*	GC 1703-117	MZ422785	MZ637177	China	Chen et al. (2021)
*Phanerochaete affinis*	KHL 11839	EU118652	EU118652	Sweden	Larsson (2007)
*Phanerochaete albida*	WEI 18-365	MZ422789	MZ637180	China	Chen et al. (2021)
*Phanerochaete albida*	GC 1407-14	MZ422788	MZ637179	China	Chen et al. (2021)
*Phanerochaete albocremea*	CLZhao 31998	PQ454009	PQ454675	China	Xu et al. (2025)
*Phanerochaete albocremea*	CLZhao 32035	PQ454011	PQ454677	China	Xu et al. (2025)
***Phanerochaete albomarginata***	CLZhao 47454*	**PZ261653**	**PZ356940**	**China**	**Present study**
*Phanerochaete alnea*	Larsson 12054	KX538924	—	Norway	Spirin et al. (2017)
*Phanerochaete alpina*	Wu 1308-61*	MZ422790	MZ637182	China	Chen et al. (2021)
*Phanerochaete alpina*	Wu 1308-77	MZ422791	MZ637183	China	Chen et al. (2021)
*Phanerochaete amphibolia*	KHL 8145	PV197698	—	Russia	Larsson et al. (2025)
*Phanerochaete arizonica*	RLG-10248-Sp*	KP135170	KP135239	USA	Floudas and Hibbett (2015)
*Phanerochaete australis*	He 6013	MT235656	MT248136	China	Luo et al. (2024)
*Phanerochaete australis*	HHB-7105-Sp	KP135081	KP135240	USA	Floudas and Hibbett (2015)
*Phanerochaete australosanguinea*	MA: Fungi: 91308	MH233925	MH233928	Chile	Luo et al. (2024)
*Phanerochaete bambusicola*	He 3606	MT235657	MT248137	China	Xu et al. (2020)
*Phanerochaete bambusicola*	Wu 0707-2*	MF399404	MF399395	China	Wu et al. (2018)
*Phanerochaete brunnea*	He 4192	MT235658	MT248138	China	Xu et al. (2020)
*Phanerochaete burdsallii*	He 2066*	MT235690	MT248177	USA	Xu et al. (2020)
*Phanerochaete burtii*	HHB-4618	KP135117	KP135241	USA	Floudas and Hibbett (2015)
*Phanerochaete burtii*	FD-171	KP135116	—	USA	Floudas and Hibbett (2015)
*Phanerochaete cacao*	IM 16–08-2006	PV197691	PV197691	Sweden	Larsson et al. (2025)
*Phanerochaete cacao*	OM 6014004	PV197692	PV197692	Finland	Larsson et al. (2025)
*Phanerochaete calotricha*	VS 7701	PV197693	—	Russia	Larsson et al. (2025)
*Phanerochaete calotricha*	KHL 14845	PV197695	—	Sweden	Larsson et al. (2025)
*Phanerochaete canobrunnea*	He 5726	MT235659	MT248139	Sri Lanka	Luo et al. (2024)
*Phanerochaete canobrunnea*	CHWC 150666	LC412095	LC412104	China	Luo et al. (2024)
*Phanerochaete canolutea*	TNM F14823	NR175166	NG153829	China	Chen et al. (2021)
*Phanerochaete carnosa*	He 5172	MT235660	MT248140	China	Xu et al. (2020)
*Phanerochaete carnosa*	HHB-9195	KP135129	KP135242	USA	Floudas and Hibbett (2015)
*Phanerochaete castanea*	Dai 24915*	PP960566	PP960569	China	Luo et al. (2024)
*Phanerochaete castanea*	Dai 24916	PP960567	PP960570	China	Luo et al. (2024)
*Phanerochaete chrysosporium*	He 5778	MT235661	MT248141	Sri Lanka	Xu et al. (2020)
*Phanerochaete chrysosporium*	HHB-6251-Sp*	KP135094	KP135246	USA	Floudas and Hibbett (2015)
*Phanerochaete cinerea*	He 5998*	—	MT248171	China	Xu et al. (2020)
*Phanerochaete cinerea*	He 6003	—	MT248172	China	Xu et al. (2020)
*Phanerochaete citrinoalba*	Dai 26584*	PP779892	PP779887	China	Luo et al. (2024)
*Phanerochaete citrinorhizomorpha*	Dai 20753	PP960568	PP960571	China	Luo et al. (2024)
*Phanerochaete citrinorhizomorpha*	Dai 26417*	PP779891	PP779886	China	Luo et al. (2024)
*Phanerochaete citrinosanguinea*	FP-105385-Sp	KP135100	—	USA	Floudas and Hibbett (2015)
*Phanerochaete citrinosanguinea*	FD-287*	KP135095	—	USA	Floudas and Hibbett (2015)
*Phanerochaete concrescens*	He 4657	MT235662	MT248142	China	Chen et al. (2021)
*Phanerochaete concrescens*	Spirin 7322*	KP994380	KP994382	Russia	Volobuev et al. (2015)
*Phanerochaete conifericola*	OM 8110	KP135171	—	Finland	Floudas and Hibbett (2015)
*Phanerochaete crystallina*	Chen 3823	MZ422802	—	China	Chen et al. (2021)
*Phanerochaete crystallina*	Chen 3576*	MZ422801	—	China	Chen et al. (2021)
*Phanerochaete cumulodentata*	He 2995	MT235664	MT248144	China	Luo et al. (2024)
*Phanerochaete cystidiata*	He 4224	MT235665	MT248145	China	Xu et al. (2020)
*Phanerochaete cystidiata*	Wu 1708-326	LC412097	LC412100	China	Wu et al. (2018)
*Phanerochaete ericina*	HHB-2288	KP135167	KP135247	USA	Floudas and Hibbett (2015)
*Phanerochaete ericina*	He 4285	MT235666	MT248146	China	Xu et al. (2020)
*Phanerochaete fissurata*	CLZhao 35311*	PQ454013	PQ454678	China	Xu et al. (2025)
*Phanerochaete fissurata*	CLZhao 35321	PQ454014	PQ454679	China	Xu et al. (2025)
*Phanerochaete fusca*	Wu 1409-163	LC412099	LC412106	China	Wu et al. (2018)
*Phanerochaete fusca*	Wu 1409-161*	LC412098	LC412105	China:	Wu et al. (2018)
*Phanerochaete granulata*	Chen 2835	MZ422808	MZ637194	China	Chen et al. (2021)
*Phanerochaete granulata*	Wu 9210-57*	MZ422810	MZ637196	China	Chen et al. (2021)
*Phanerochaete guangdongensis*	Wu 1809-348*	MZ422813	MZ637199	China	Chen et al. (2021)
*Phanerochaete guangdongensis*	Wu 1809-319	MZ422811	MZ637197	China	Chen et al. (2021)
*Phanerochaete hainanensis*	He 3562*	MT235692	MT248179	China	Li et al. (2023)
*Phanerochaete hymenochaetoides*	He 5988*	—	MT248173	China	Xu et al. (2020)
*Phanerochaete incarnata*	He 20120728-1	MT235669	MT248149	China	Xu et al. (2020)
*Phanerochaete incarnata*	WEI 16-075	MF399406	MF399397	China	Wu et al. (2018)
*Phanerochaete krikophora*	HHB 5796	KP135164	KP135268	USA	Floudas and Hibbett (2015)
*Phanerochaete laevis*	He 20120917-8	MT235670	MT248150	China	Xu et al. (2020)
*Phanerochaete laevis*	HHB-15519	KP135149	KP135249	USA	Floudas and Hibbett (2015)
*Phanerochaete leptocystidiata*	He 5853*	MT235685	MT248168	China	Xu et al. (2020)
*Phanerochaete leptocystidiata*	Dai 10468	MT235684	MT248167	China	Xu et al. (2020)
*Phanerochaete livescens*	He 5010	MT235671	MT248151	China	Luo et al. (2024)
*Phanerochaete metuloidea*	He 2766	MT235682	MT248164	China	Xu et al. (2020)
*Phanerochaete minor*	He 3988*	MT235686	MT248170	China	Xu et al. (2020)
*Phanerochaete mopanshanensis*	CLZhao 2357*	OR096190	OR461450	China	Dong et al. (2024)
***Phanerochaete nigella***	CLZhao 48735*	**PZ261654**	**PZ356941**	**China**	**Present Study**
***Phanerochaete nigella***	**CLZhao 50724**	**PZ356947**	**PZ356942**	**China**	**Present Study**
*Phanerochaete ochraceofulva*	KHL 15178	PV197706	PV197706	Spain	Larsson et al. (2025)
*Phanerochaete parmastoi*	He 4570	MT235673	MT248153	China	Xu et al. (2020)
*Phanerochaete parmastoi*	Wu 880313-6*	MZ422823	GQ470654	China	Chen et al. (2021)
*Phanerochaete porostereoides*	He1902	KX212217	KX212221	China	Chen et al. (2021)
*Phanerochaete porostereoides*	He1908*	KX212218	KX212222	China	Chen et al. (2021)
*Phanerochaete pruinosa*	CLZhao 7112	MZ435346	MZ435350	China	Wang and Zhao (2021)
*Phanerochaete pruinosa*	CLZhao 7113*	MZ435347	MZ435351	China	Wang and Zhao (2021)
*Phanerochaete pseudosanguinea*	FD-244*	KP135098	KP135251	USA	Floudas and Hibbett (2015)
*Phanerochaete punctata*	CLZhao 30365	PQ454015	PQ454680	China	Xu et al. (2025)
*Phanerochaete punctata*	CLZhao 30512*	PQ454016	PQ454681	China	Xu et al. (2025)
*Phanerochaete rhizomorpha*	GC 1708-335*	MZ422824	MZ637208	China	Chen et al. (2021)
*Phanerochaete rhizomorpha*	GC 1708-354	MZ422825	MZ637209	China	Chen et al. (2021)
*Phanerochaete rhodella*	FD-18	KP135187	KP135258	USA	Floudas and Hibbett (2015)
*Phanerochaete rufobrunnea*	LE292081	PV197688	—	Russia	Larsson et al. (2025)
*Phanerochaete sanguineocarnosa*	FD-359	KP135122	KP135245	USA	Floudas and Hibbett (2015)
*Phanerochaete sinensis*	He4660*	MT235688	MT248175	China	Xu et al. (2020)
*Phanerochaete sinensis*	GC1809-56	MT235689	MT248176	China	Xu et al. (2020)
*Phanerochaete sordida*	FD-241	KP135136	KP135252	USA	Floudas and Hibbett (2015)
*Phanerochaete spadicea*	Wu 0504-15*	MZ422837	MZ637219	China	Chen et al. (2021)
*Phanerochaete spadicea*	Wu 0504-11	MZ422836	—	China	Chen et al. (2021)
*Phanerochaete stereoides*	He 5824	MT235677	MT248158	Sri Lanka	Xu et al. (2020)
*Phanerochaete stereoides*	He 2309	KX212219	KX212223	China	Liu and He (2016)
*Phanerochaete subcarnosa*	Wu 9310-3*	MZ422841	GQ470642	China	Chen et al. (2021)
*Phanerochaete subcarnosa*	GC 1809-90	MZ422840	MZ637222	China	Chen et al. (2021)
*Phanerochaete subrosea*	He 2421*	MT235687	MT248174	China	Xu et al. (2020)
*Phanerochaete subsanguinea*	CLZhao 10477*	MZ435349	MZ435353	China	Wang and Zhao (2021)
*Phanerochaete subsanguinea*	CLZhao 10470	MZ435348	MZ435352	China	Wang and Zhao (2021)
*Phanerochaete subtropica*	CLZhao F8716*	OP605486	OQ195089	China	Yu et al. (2023)
*Phanerochaete subtropica*	CLZhao F2763	OP605518	OQ195090	China	Yu et al. (2023)
*Phanerochaete subtuberculata*	CLZhao F5130	OP605484	OQ195088	China	Yu et al. (2023)
*Phanerochaete subtuberculata*	CLZhao F6838*	OP605485	OQ195087	China	Yu et al. (2023)
*Phanerochaete taiwaniana*	He 5269	MT235680	MT248161	Vietnam	Xu et al. (2020)
*Phanerochaete taiwaniana*	Wu 0112-13	MF399412	MF399403	China	Chen et al. (2021)
*Phanerochaete velutina*	2025CLZhao 85	PZ356948	—	Belgium	Unpublished
*Phanerochaete velutina*	2025CLZhao 110	PZ356949	—	Belgium	Unpublished
*Phanerochaete velutina*	GC 1604-56	MZ422844	MZ637224	China	Chen et al. (2021)
*Phanerochaete velutina*	He 3079	MT235681	MT248162	China	Xu et al. (2020)
*Phanerochaete velutina*	Kotiranta 25567	KP994354	KP994387	Russia	Volobuev et al. (2015)
*Phanerochaete volubilis*	VS 5465	PV197707	PV197707	Russia	Larsson et al. (2025)
*Phanerochaete volubilis*	VS 4869	PV197708	PV197708	Russia	Larsson et al. (2025)
*Phanerochaete wuyiensis*	Dai 25530*	PP779888	PP779883	China	Luo et al. (2024)
*Phanerochaete wuyiensis*	Dai 26250	PP779890	PP779885	China	Luo et al. (2024)
*Phanerochaete xizangensis*	LWZ 20230716-8b	PP959665	PP959652	China	Wang et al. (2024)
*Phanerochaete xizangensis*	LWZ 20230714-22b*	PP959664	PP959651	China	Wang et al. (2024)
*Phanerochaete yingjiangensis*	CLZhao 37439	PV820772	—	China	Wijesinghe et al. (2025)
*Phanerochaete yingjiangensis*	CLZhao 37443*	PV820773	—	China	Wijesinghe et al. (2025)
*Phanerochaete yunnanensis*	He 2719*	MT235683	MT248166	China	Xu et al. (2020)
*Phanerochaete yunnanensis*	He 2697	—	MT248165	China	Xu et al. (2020)
*Phlebiopsis alba*	GC 1708-20	MZ637043	MZ637247	China	Chen et al. (2021)
*Phlebiopsis amethystea*	URM 84741	MK993645	MK993639	Brazil	Lima et al. (2020)
*Phlebiopsis brunnea*	He 5822*	MT452527	MT447451	Sri Lanka	Li et al. (2023)
*Phlebiopsis crassa*	KKN 86	KP135394	KP135215	USA	Floudas and Hibbett (2015)
*Phlebiopsis crassa*	MAFF 420737	AB809163	AB809163	Japan	Takano et al. (2006)
*Phlebiopsis flavidoalba*	KHL 13055	EU118662	EU118662	Costa Rica	Larsson (2007)
*Phlebiopsis galochroa*	FP-102937	KP135391	KP135270	USA	Justo et al. (2017)
*Phlebiopsis gigantea*	FBCC 315	LN611131	LN611131	Sweden	Kuuskeri et al. (2015)
*Phlebiopsis gigantea*	FP-70857	KP135390	KP135272	USA	Floudas and Hibbett (2015)
*Phlebiopsis laxa*	Wu 9311-17*	MT561710	GQ470649	China	Wu et al. (2010)
*Phlebiopsis odontoidea*	GC 1708-181*	MZ637054	MZ637255	China	Chen et al. (2021)
*Pirex concentricus*	Kropp160Bup6-R	KP134985	—	USA	Floudas and Hibbett (2015)
*Pirex concentricus*	OSC-41587	KP134984	KP135275	USA	Floudas and Hibbett (2015)
*Porostereum fulvum*	LY:18491	MG649452	MG649454	France	Unpublished
*Porostereum spadiceum*	Wu 9508-139	MZ637062	MZ637263	China	Chen et al. (2021)
*Quasiphlebia densa*	WEI 17-057*	MZ637066	MZ637265	USA	Chen et al. (2021)
*Quasiphlebia densa*	Wu 9304-33	MZ637067	MZ637266	China	Chen et al. (2021)
*Rhizochaete americana*	FP-102188	KP135409	KP135277	USA	Floudas and Hibbett (2015)
*Rhizochaete americana*	HHB 2004	AY219391	AY219391	USA	Greslebin et al. (2004)
*Rhizochaete belizensis*	FP 150712	KP135408	KP135280	Belize	Floudas and Hibbett (2015)
*Rhizochaete borneensis*	WEI 16-426	MZ637070	MZ637270	China	Unpublished
*Rhizochaete brunnea*	MR 11455	AY219389	AY219389	Argentina	Greslebin et al. (2004)
*Rhizochaete chinensis*	Wu 0910-45*	LC387335	MF110294	China	Chen et al. (2018)
*Rhizochaete filamentosa*	FP 105240	KP135411	AY219393	USA	Nakasone et al. (2017)
*Rhizochaete filamentosa*	HHB 3169	KP135410	KP135278	USA	Floudas and Hibbett (2015)
*Rhizochaete fissurata*	CLZhao 10407*	MZ713642	MZ713846	China	Gu and Zhao (2021)
*Rhizochaete fissurata*	CLZhao 10418	MZ713643	MZ713847	China	Gu and Zhao (2021)
*Rhizochaete fissurata*	CLZhao 7965	MZ713641	MZ713845	China	Gu and Zhao (2021)
*Rhizochaete flava*	PR 1141	KY273030	KY273033	Puerto Rico	Nakasone et al. (2017)
*Rhizochaete flava*	PR 3148	KY273029	—	Puerto Rico	Nakasone et al. (2017)
*Rhizochaete fouquieriae*	KKN 121	AY219390	GU187608	USA	Nakasone et al. (2017)
*Rhizochaete fouquieriae*	KKN-121-sp	KY948786	KY948858	USA	Justo et al. (2017)
*Rhizochaete galactites*	Hjm 18924	OR822124	OR822124	Sweden	Larsson et al. (2025)
*Rhizochaete galactites*	VS 11261	OR822125	—	Russia	Larsson et al. (2025)
*Rhizochaete grandinosa*	CLZhao 3117*	MZ713644	MZ713848	China	Gu and Zhao (2021)
*Rhizochaete lutea*	He 5964	—	ON964008	China	Li et al. (2023)
*Rhizochaete lutea*	Wu880417-5*	MZ637072	GQ470651	China	Wu et al. (2010)
***Rhizochaete membranacea***	CLZhao 20593*	**PZ356945**	**PZ356943**	**China**	**Present study**
***Rhizochaete membranacea***	**CLZhao 50723**	**PZ356946**	**PZ356944**	**China**	**Present study**
*Rhizochaete nakasoneae*	He 2291*	ON964016	ON963998	China	Li et al. (2023)
*Rhizochaete nakasoneae*	He 4821	—	ON963999	China	Li et al. (2023)
*Rhizochaete radicata*	FD 123	KP135407	KP135279	USA	Floudas and Hibbett (2015)
*Rhizochaete radicata*	HHB 1909	AY219392	AY219392	USA	Greslebin et al. (2004)
*Rhizochaete subradicata*	He 2377	ON964023	ON964004	China	Li et al. (2023)
*Rhizochaete subradicata*	He 3213*	OP102693	OP102695	China	Li et al. (2023)
*Rhizochaete sulphurina*	DLL 2014-176	KY273032	—	USA	Nakasone et al. (2017)
*Rhizochaete sulphurina*	HHB 5604	KY273031	GU187610	USA	Nakasone et al. (2017)
*Rhizochaete sulphurosa*	KHL 16087	KT003523	—	Brazil	Chikowski et al. (2016)
*Rhizochaete sulphurosa*	URM 87190	KT003522	KT003519	Brazil	Chikowski et al. (2016)
*Rhizochaete terrestris*	He 7713*	OP549029	OP549030	China	Li et al. (2023)
*Rhizochaete terrestris*	He 6694	ON964022	ON964003	China	Li et al. (2023)
*Rhizochaete variegata*	Dai 24600*	OP874926	OP874921	China	Zhang et al. (2023)
*Rhizochaete variegata*	Dai 24601	OP874927	OP874922	China	Zhang et al. (2023)
*Rhizochaete violascens*	KHL11169	—	EU118612	Norway	Larsson (2007)
*Rhizochaete violascens*	GB-0161553	OR822123	OR822123	Sweden	Larsson (2007)
*Rhizochaete yunnanensis*	He 3302*	ON964020	ON964002	China	Li et al. (2023)
*Riopa metamorphosa*	Viacheslav Spirin 2395	KX752601	KX752601	Russia	Miettinen et al. (2016)
*Riopa pudens*	Dai 19241	OL470307	OL462822	China	Unpublished
*Roseograndinia aurantiaca*	CLZhao 10487*	MW209023	MW209012	China	Wang and Zhao (2021)
*Roseograndinia aurantiaca*	Wu 1307-132	MZ637076	MZ637274	China	Chen et al. (2021)
*Roseograndinia minispora*	WEI 18-511	MZ637079	MZ637277	China	Chen et al. (2021)
*Roseograndinia zixishanensis*	CLZhao 7206	MZ305280	MZ305289	China	Li et al. (2023)
*Roseograndinia zixishanensis*	CLZhao 7718*	MZ305285	MZ305293	China	Li et al. (2023)
*Terana coerulea*	FP-104073	KP134980	KP135276	USA	Floudas and Hibbett (2015)
*Terana coerulea*	GC 1507-2	MZ637090	MZ637287	China	Chen et al. (2021)

### Phylogenetic analyses

**Figure 1.**
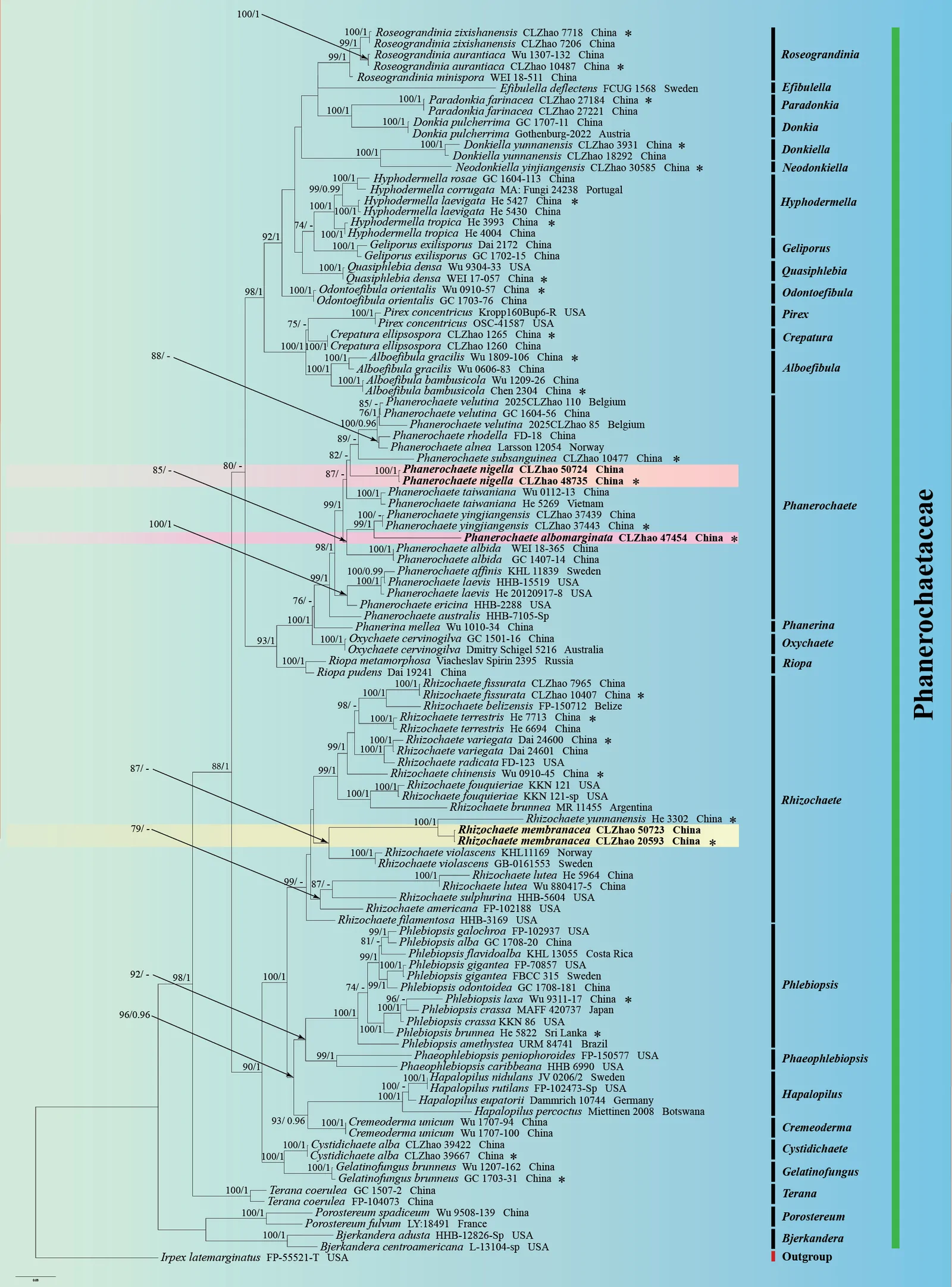
Maximum likelihood analysis illustrating the phylogeny of the three new species in *Phanerochaetaceae* based on ITS + nLSU sequences. Branches are labeled with ML bootstrap values ≥ 70% and Bayesian posterior probabilities ≥ 0.95. Colored bars represent different genera. New species are shown in bold; * refers to type material (holotype).

**Figure 2.**
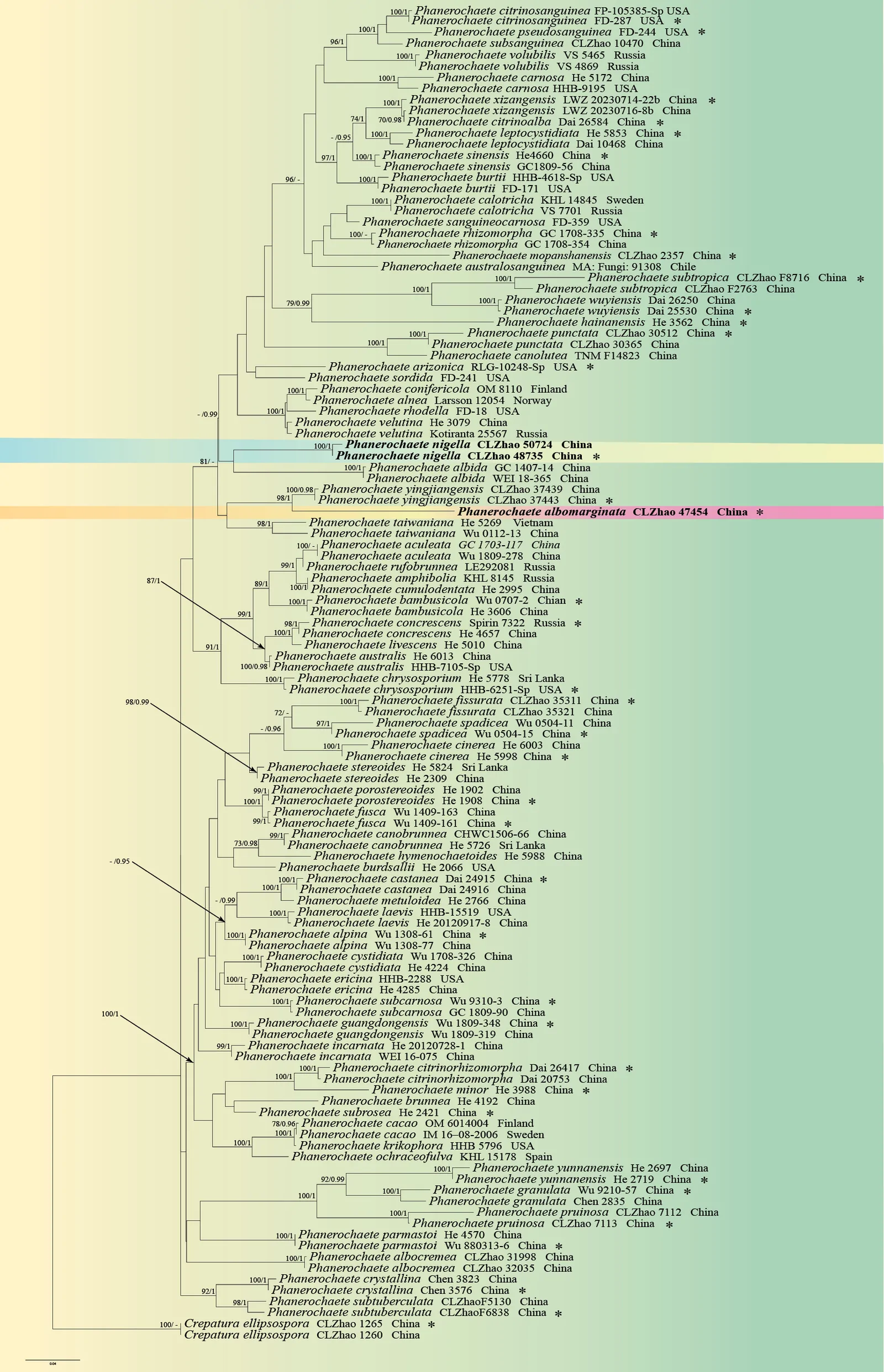
Maximum likelihood analysis illustrating the phylogeny of the two new species in *Phanerochaete* based on ITS + nLSU sequences. Branches are labeled with ML bootstrap values ≥ 70% and Bayesian posterior probabilities ≥ 0.95. Colored bars represent different genera. New species are shown in bold; * refers to type material (holotype).

**Figure 3.**
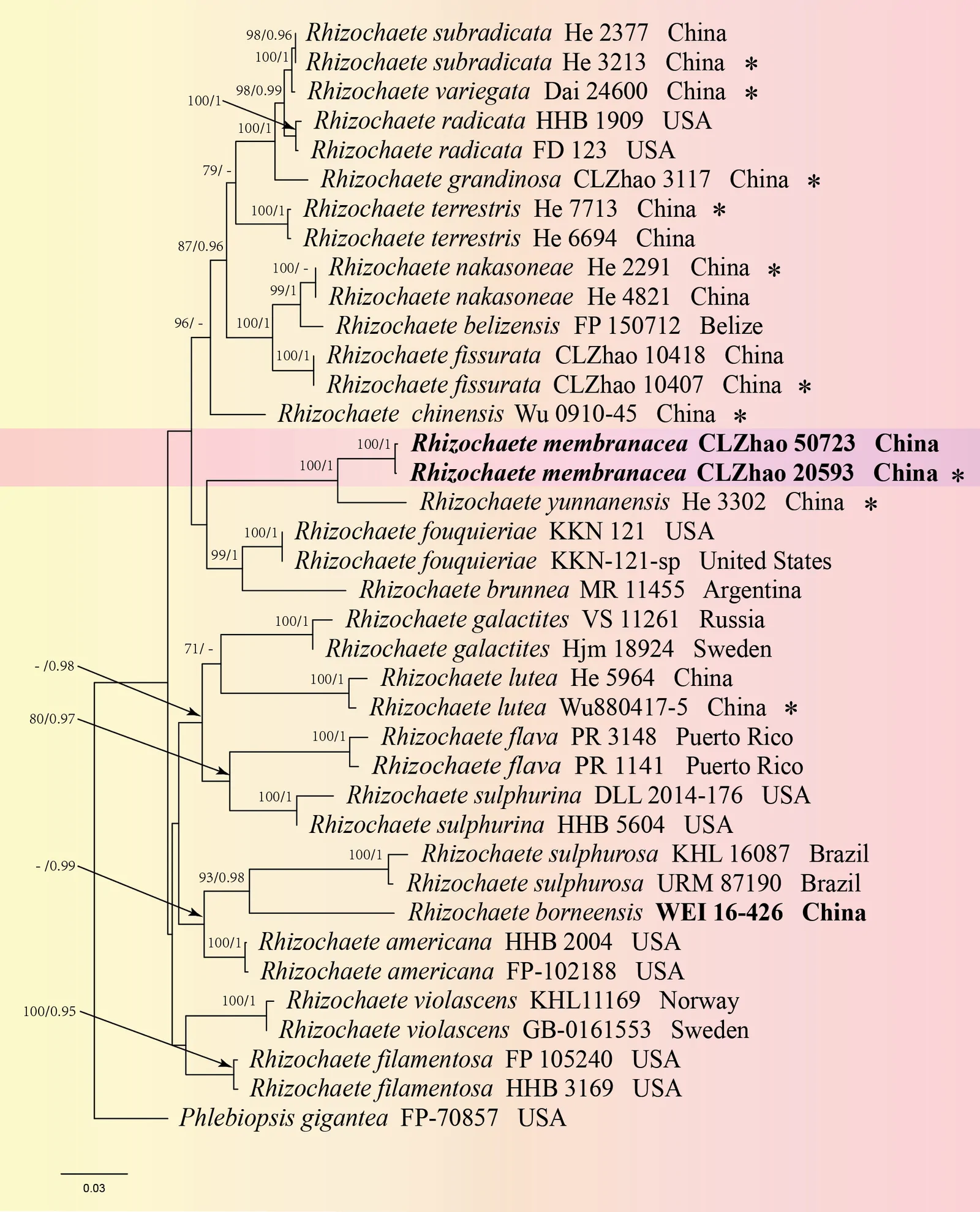
Maximum likelihood analysis illustrating the phylogeny of the new species in *Rhizochaete* based on ITS + nLSU sequences. Branches are labeled with ML bootstrap values ≥ 70% and Bayesian posterior probabilities ≥ 0.95. Colored bars represent different genera. New species are shown in bold; * refers to type material (holotype).

Sequences generated for this study were aligned with additional sequences downloaded from GenBank, and all nucleotide sequences are systematically listed in Table 1. The sequences were aligned with MAFFT version 7 (Katoh et al. 2019) using the G-INS-i strategy. The alignment was adjusted manually using AliView version 1.27 (Larsson 2014). Each dataset was first aligned separately, and then the ITS + nLSU regions were combined with Mesquite version 3.51. For this analysis, sequences of *Irpex latemarginatus* (Durieu & Mont.) C.C. Chen & Sheng H. Wu were selected as the outgroup in the ITS + nLSU analysis (Fig. 1), following Zhang et al. (2026). Sequences of *Crepatura ellipsospora* C.L. Zhao were selected as the outgroup in the ITS + nLSU analysis (Fig. 2), following a previous study (Chen et al. 2021). Sequences of *Phlebiopsis gigantea* (Fr.) Jülich were selected as the outgroup in the ITS + nLSU analysis (Fig. 3), following a previous study (Li et al. 2023).

Maximum likelihood (ML) and Bayesian inference (BI) analyses were performed on the combined ITS + nLSU datasets, following a previous study (Luo et al. 2024). ML analysis was performed using RAxML-HPC BlackBox on the CIPRES Science Gateway (https://www.phylo.org/portal2/login!input.action; Miller et al. 2012) with a GTRCAT model of evolution and 1,000 bootstrap replicates. ModelFinder v2.2.0 (Kalyaanamoorthy et al. 2017) was used to select the best-fit model using the Bayesian information criterion (BIC). BI phylogenies were inferred using MrBayes v3.2.7a (Ronquist et al. 2012) as implemented in PhyloSuite v1.2.3 (Zhang et al. 2020; Xiang et al. 2023), and branches were deemed significantly supported when they received an ML bootstrap value (BS) of ≥ 70% or a Bayesian posterior probability (BPP) of ≥ 0.95.

## Results

### Phylogenetic analyses

The ITS + nLSU dataset (Fig. 1) included sequences from 110 fungal specimens representing 57 species belonging to 29 genera in the families *Phanerochaetaceae* and *Rhizochaetaceae*. The best-fit model estimated for the ITS + nLSU alignment and applied in BI was GTR+F+I+G4 (2 parallel runs, 3,000,000 generations). At the end of the BI runs, the average standard deviation of split frequencies was 0.007359, and the effective sample size (ESS) across the two runs was twice the average ESS (avg. ESS = 900.5). The tree topology obtained by BI was similar to that obtained by ML. The phylogram based on the combined ITS + nLSU sequences (Fig. 1) revealed that *Phanerochaete albomarginata* and *P. nigella* were grouped within the genus *Phanerochaete*, while *Rhizochaete membranacea* was grouped within the genus *Rhizochaete*.

The aligned ITS + nLSU dataset (Fig. 2) comprised 120 specimens representing 73 species. The best-fit model estimated for the ITS + nLSU alignment and applied in BI was GTR+I+G (two parallel runs, 3,000,000 generations). At the end of the BI runs, the average standard deviation of split frequencies was 0.010323, and the effective sample size (ESS) across the two runs was twice the average ESS (avg. ESS = 694.5). The phylogenetic tree (Fig. 2), inferred from ITS + nLSU sequences, showed that *Phanerochaete albomarginata* was closely related to *P. taiwaniana* Sheng H. Wu and *P. yingjiangensis* W.Y. Xiao & H.M. Zhou, while *P. nigella* formed a sister lineage to *P. albida* Sheng H. Wu. Thus, *P. albomarginata* and *P. nigella* are introduced as new taxa collected from Yunnan Province, China.

The aligned ITS + nLSU dataset (Fig. 3) comprised 38 specimens representing 23 species. The best-fit model estimated for the ITS + nLSU alignment and applied in BI was GTR+F+I+G4 (2 parallel runs, 1,000,000 generations). At the end of the BI runs, the average standard deviation of split frequencies was 0.007274, and the effective sample size (ESS) across the two runs was twice the average ESS (avg. ESS = 679). The topology based on ITS + nLSU sequences (Fig. 3) revealed that *Rhizochaete membranacea* formed a separate lineage sister to *R. yunnanensis* Yue Li & S.H. He, with 100% BS and 1.00 BPP. Thus, *R. membranacea* is proposed as a new species.

### Taxonomy

#### Phanerochaete albomarginata

Taxon classificationFungiPolyporalesPhanerochaetaceae

Q. Li & C.L. Zhao sp. nov.

556575F7-1FB7-5F83-B465-7C02A624C2FF

863734

Figs 4, 5, 6

##### Holotype.

China, • Yunnan Province, Puer, Jinggu County, Weiyuanjiang Provincial Nature Reserve, 23°11'N, 100°33'E, elevation 1,005 m asl., on fallen angiosperm branch, leg. C.L. Zhao, 3 October 2025, CLZhao 47454 (SWFC 00047454).

**Figure 4.**
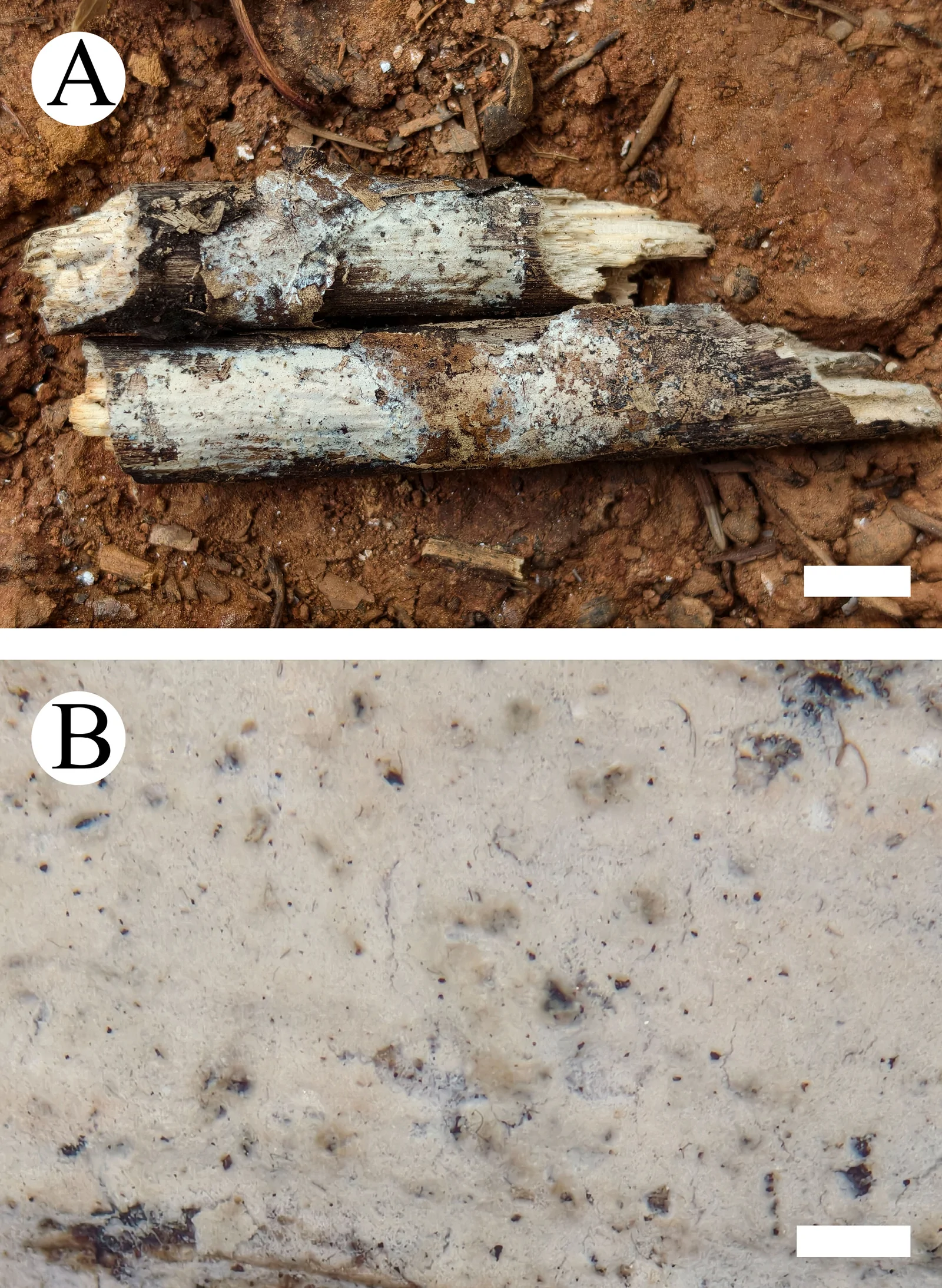
Basidiomata of *Phanerochaete albomarginata* (SWFC 00047454, holotype). Scale bars: 1 cm (**A**); 1 mm (**B**).

**Figure 5.**
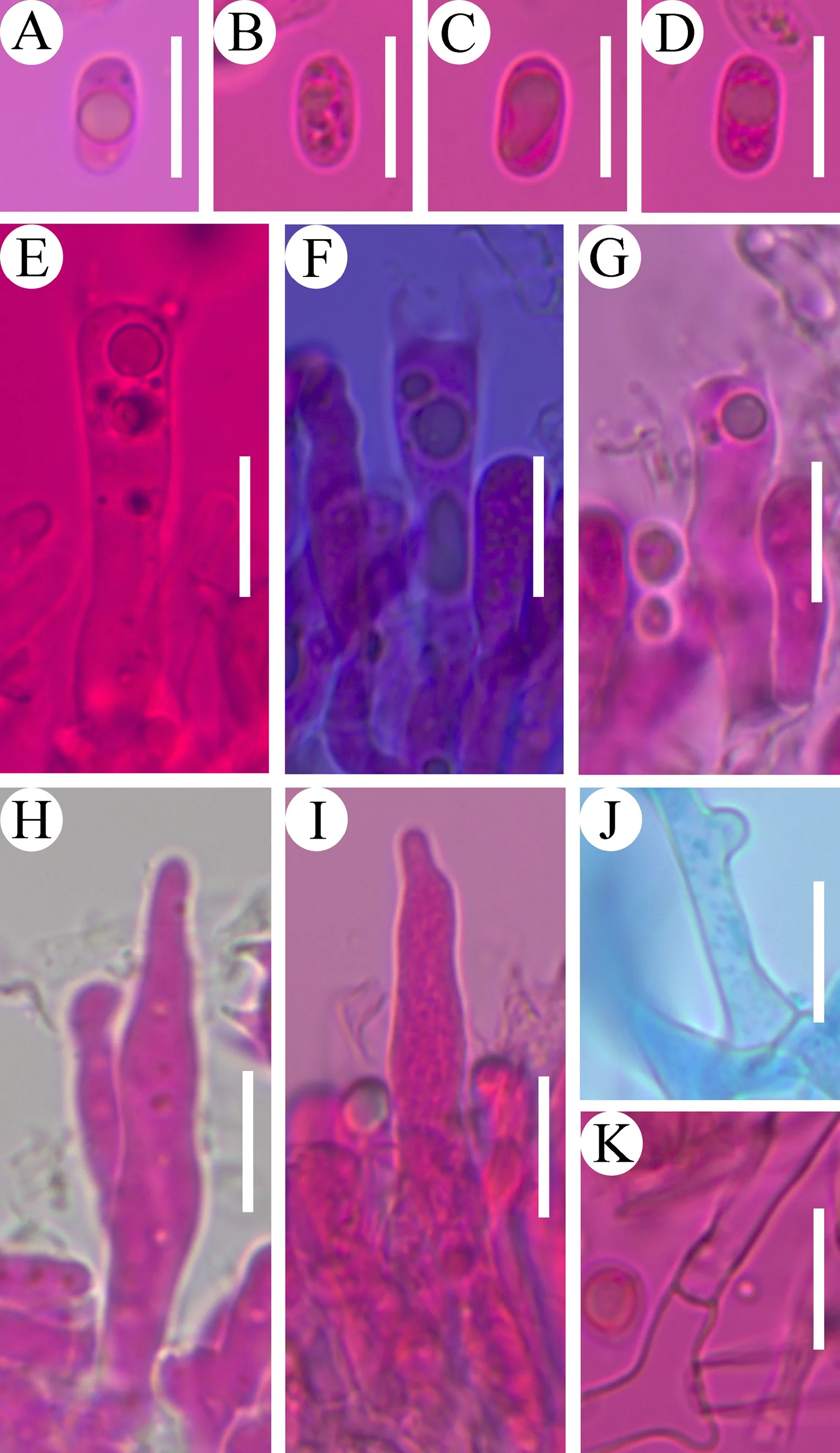
Sections of the hymenium of *Phanerochaete albomarginata* (SWFC 00047454, holotype). **A–D**. Basidiospores; **E–G**. Basidia; **H, I**. Cystidia; **J, K**. Generative hyphae. Scale bars: 10 µm (**A–K**); 10 × 100 oil.

**Figure 6.**
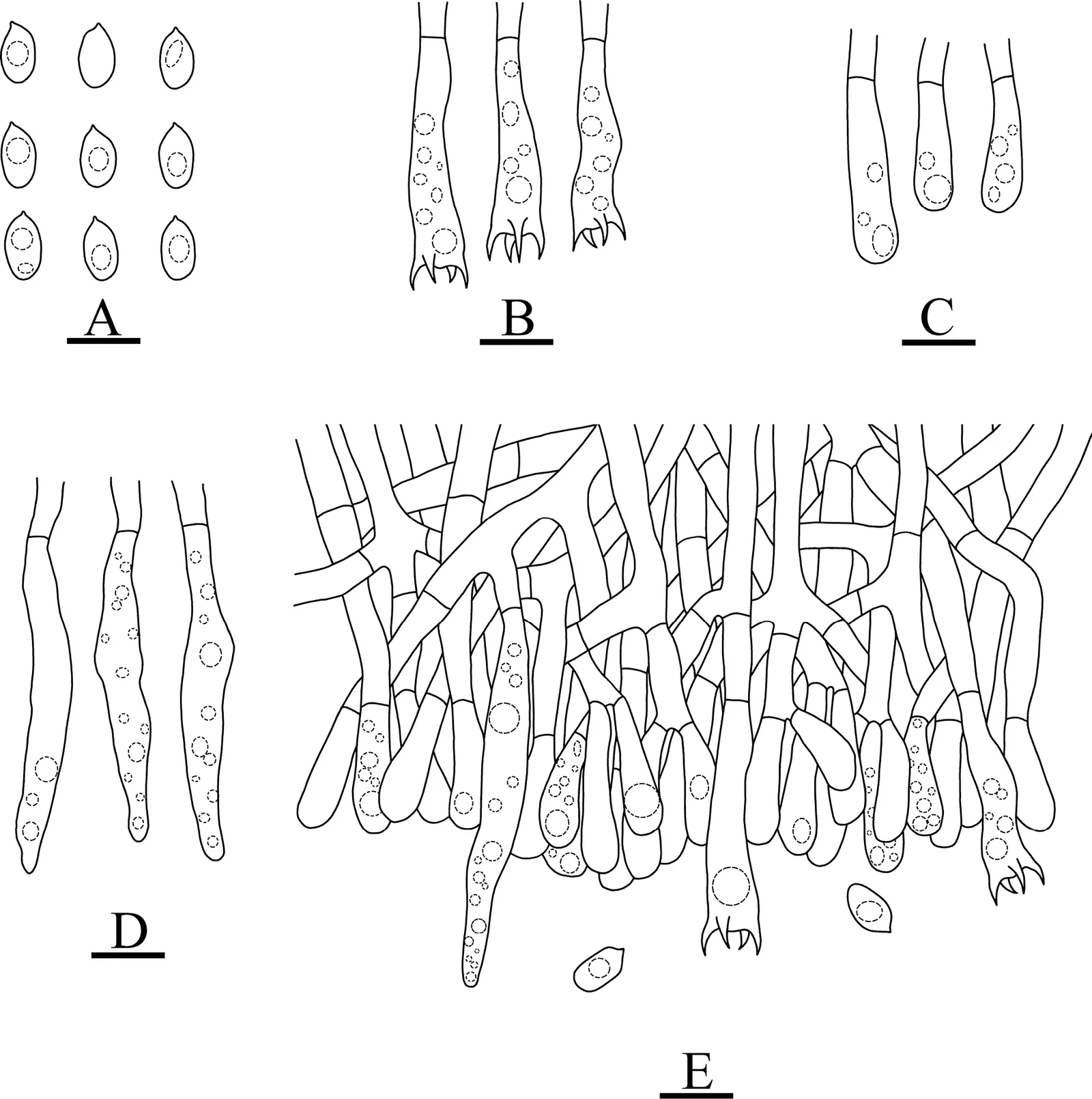
Microscopic structures of *Phanerochaete albomarginata* (SWFC 00047454, holotype). **A**. Basidiospores; **B**. Basidia; **C**. Basidioles; **D**. Cystidia; **E**. Part of a vertical section of the hymenium. Scale bars: 10 μm (**A–E**).

##### Etymology.

***Albomarginata*** (Lat.) refers to the white margin of the type specimens.

##### Basidiomata.

Annual, resupinate, closely adnate, membranaceous, without odor or taste when fresh, becoming fragile upon drying, up to 4 cm long, 2 cm wide, 150 µm thick. Hymenial surface smooth, cream when fresh, cream to buff upon drying. Sterile margin white, narrow, up to 1 mm wide.

##### Hyphal structure.

Monomitic, generative hyphae with simple septate, colorless, thin-walled, branched, interwoven, 2.5–6.5 µm in diameter, IKI–, CB–; tissues unchanged in KOH.

##### Hymenium.

Cystidia abundant, embedded or slightly projecting, tapering, distinctly thin-walled, occasionally with oil drops, without crystals, 27–59 × 5–6.5 µm. Basidia clavate, occasionally with oil drops, with four sterigmata and a basal simple septum, 25–32 × 6–7.5 µm; basidioles dominant, occasionally with oil drops, similar to basidia in shape, but slightly smaller.

##### Basidiospores.

Ellipsoid, colorless, smooth, thin-walled, often with oily drops, IKI–, CB–, (6.8–)7.1–9.2(–9.4) × (4–)4.1–4.9(–5.3) µm, *L* = 8.07 µm, *W* = 4.60 µm, *Qm* = 1.76 ± 0.14 (*n* = 30/1).

##### Type of rot.

White rot.

#### Phanerochaete nigella

Taxon classificationFungiPolyporalesPhanerochaetaceae

Q. Li & C.L. Zhao sp. nov.

AEDB0D8A-976C-51ED-98F6-CD4527472189

863741

Figs 7, 8, 9

##### Holotype.

China, • Yunnan Province, Puer, Jinggu County, Mangyu Grand Canyon, 23°53'N, 100°26'E, elevation 1,200 m asl., on fallen angiosperm branch, leg. C.L. Zhao, 6 October 2025, CLZhao 48735 (SWFC 00048735).

**Figure 7.**
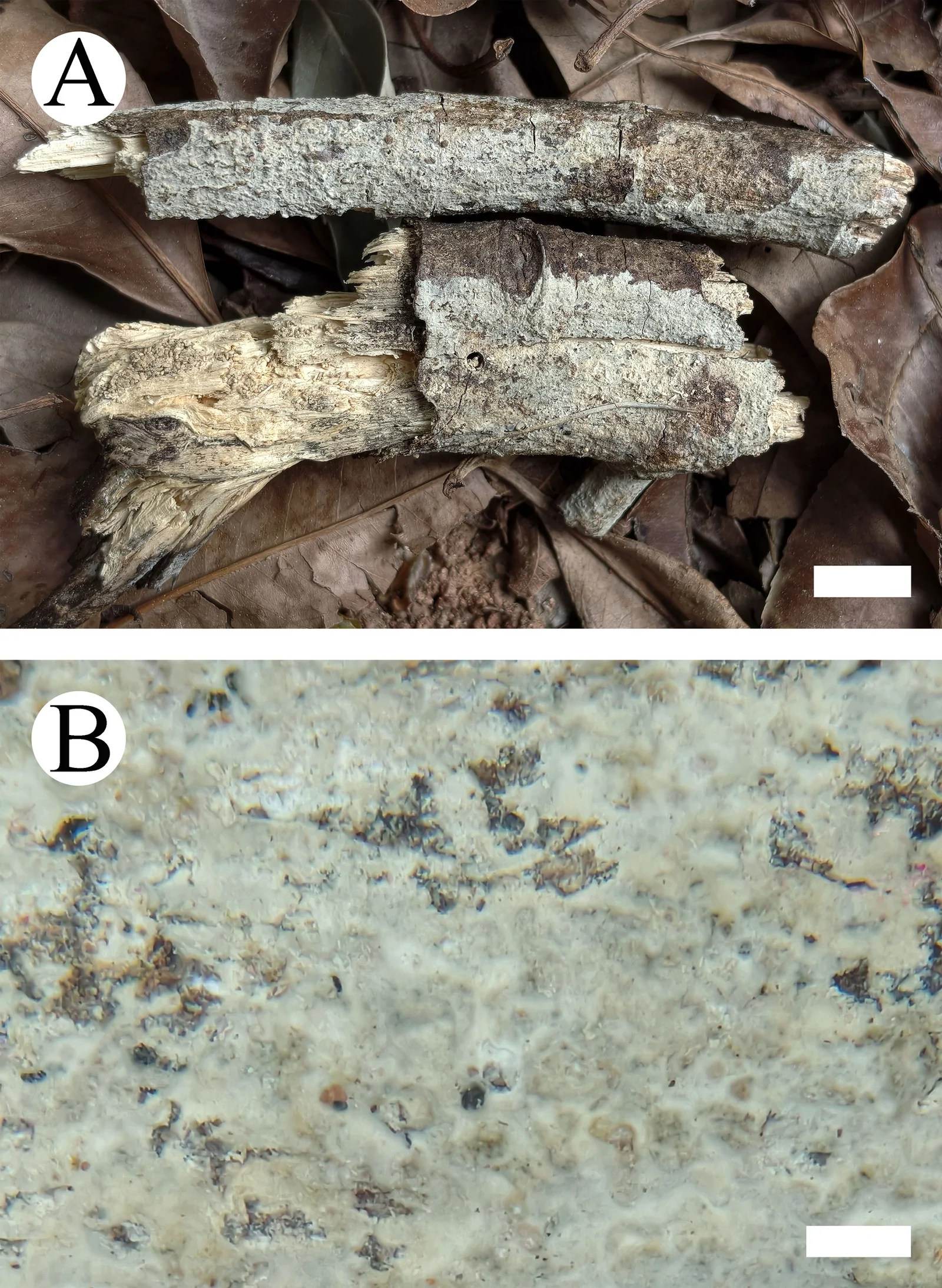
Basidiomata of *Phanerochaete nigella* (SWFC 00048735, holotype). Scale bars: 1 cm (**A**); 1 mm (**B**).

**Figure 8.**
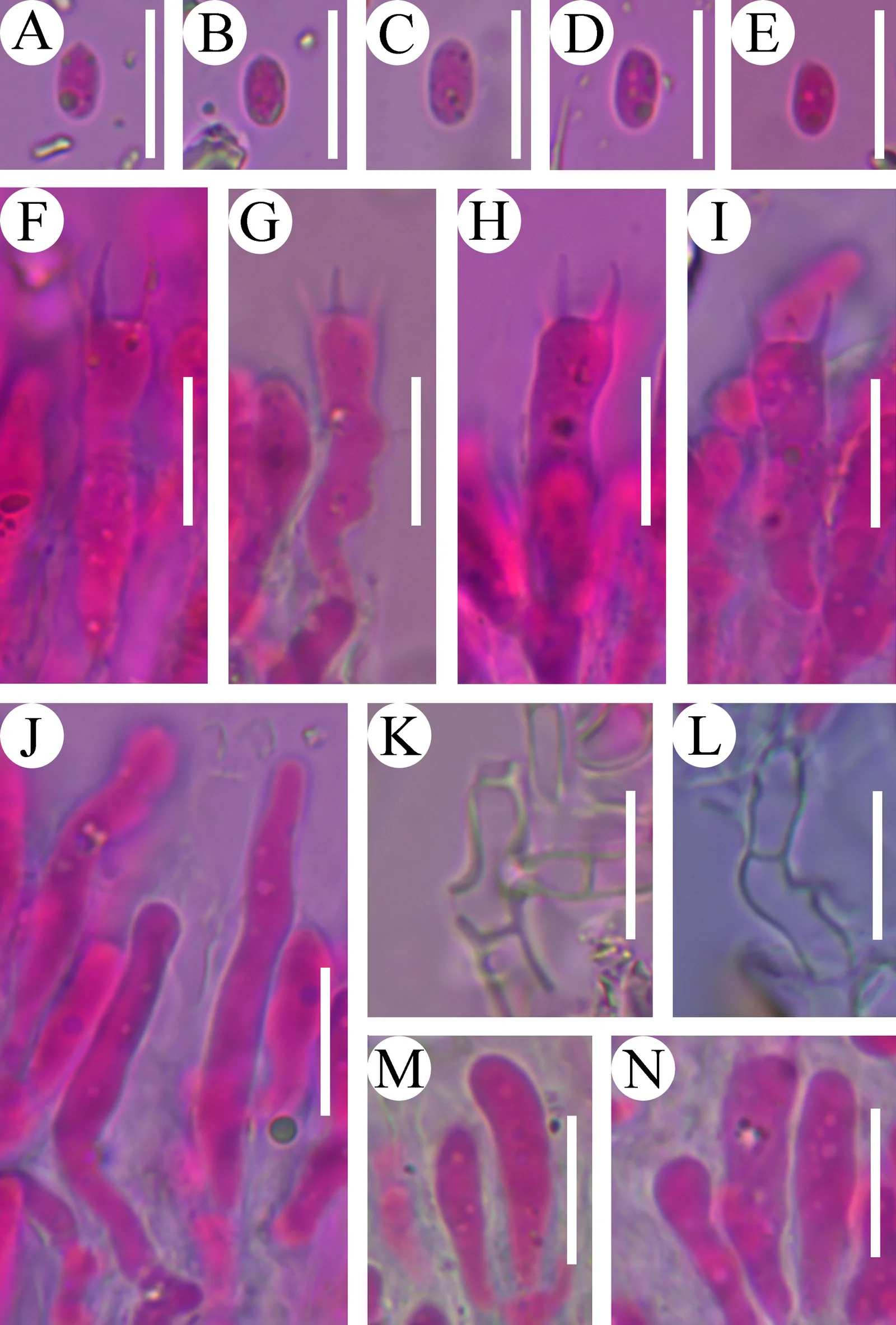
Sections of the hymenium of *Phanerochaete nigella* (SWFC 00048735, holotype). **A–E**. Basidiospores; **F–I**. Basidia; **J**. Cystidia; **K, L**. Generative hyphae; **M, N**. Basidioles. Scale bars: 10 µm (**A–N**); 10 × 100 oil.

**Figure 9.**
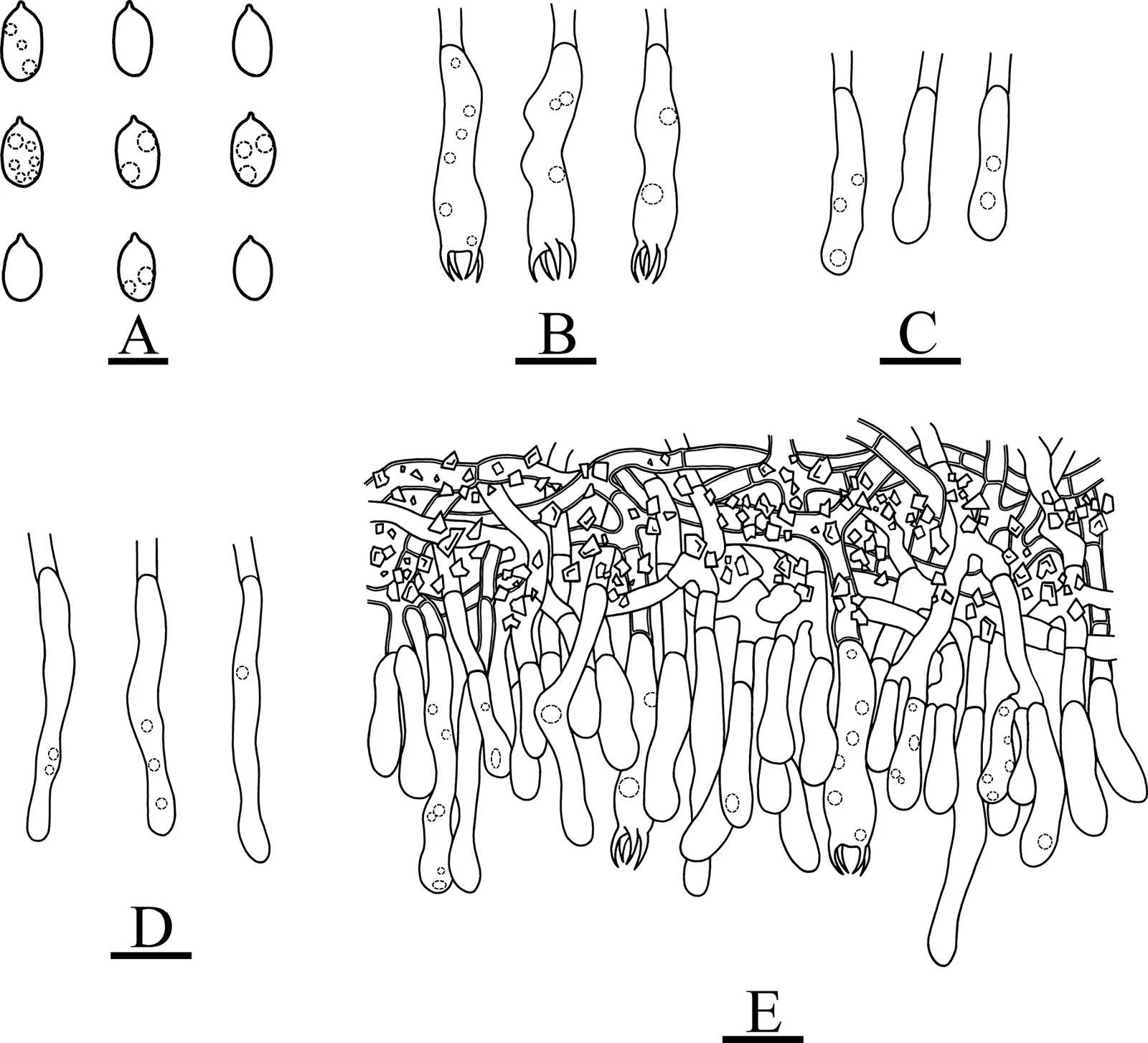
Microscopic structures of *Phanerochaete nigella* (SWFC 00048735, holotype). **A**. Basidiospores; **B**. Basidia; **C**. Basidioles; **D**. Cystidia; **E**. Part of a vertical section of the hymenium. Scale bars: 5 μm (**A**); 10 μm (**B–E**).

##### Etymology.

*Nigella* (Lat.) refers to the black hymenial surface of the type specimens.

##### Basidiomata.

Annual, resupinate, adnate, coriaceous, without odor or taste when fresh, becoming fragile upon drying, up to 8 cm long, 4 cm wide, 100 µm thick. Hymenial surface tuberculate, gray when fresh, gray to black upon drying. Sterile margin grayish-white, narrow, up to 0.5 mm wide.

##### Hyphal structure.

Monomitic, generative hyphae with simple septate, colorless, numerous crystals present among hyphae, thin- to thick-walled, branched, interwoven, 2–4 µm in diameter, IKI–, CB–; tissues unchanged in KOH.

##### Hymenium.

Cystidia abundant, tubular, slightly flexuous, thin-walled, embedded in subhymenium or exserted, 23–47 × 3–4.5 µm. Basidia clavate, slightly flexuous, occasionally with oil drops, with four sterigmata and a basal simple septum, 15–25 × 4.5–5.5 µm; basidioles dominant, occasionally with oil drops, similar to basidia in shape, but slightly smaller.

##### Basidiospores.

Ellipsoid, colorless, smooth, thin-walled, often with oily drops, IKI–, CB–, (4.5–)4.7–5.7(–5.9) × 2.5–3.2 µm, *L* = 5.20 µm, *W* = 2.90 µm, *Qm* = 1.61 ± 0.18 (*n* = 60/2).

##### Type of rot.

White rot.

##### Additional specimen

**(paratype) examined**. China, • Yunnan Province, Puer, Jinggu County, Mangyu Grand Canyon, 23°53'N, 100°26'E, elevation 1,200 m asl., on fallen angiosperm branch, leg. C.L. Zhao, 15 November 2025, CLZhao 50724 (SWFC 00050724).

#### Rhizochaete membranacea

Taxon classificationFungiPolyporalesPhanerochaetaceae

Q. Li & C.L. Zhao sp. nov.

99F6FCE5-CB17-5E75-B1D5-17B4072B3882

863744

Figs 10, 11, 12

##### Holotype.

China, • Yunnan Province, Zhaotong, Qiaojia County, Yaoshan Town, Yaoshan National Nature Reserve, 27°11'N, 103°07'E, elevation 3251 m asl., on fallen angiosperm branch, leg. C.L. Zhao, 22 August 2020, CLZhao 20593 (SWFC 00020593).

**Figure 10.**
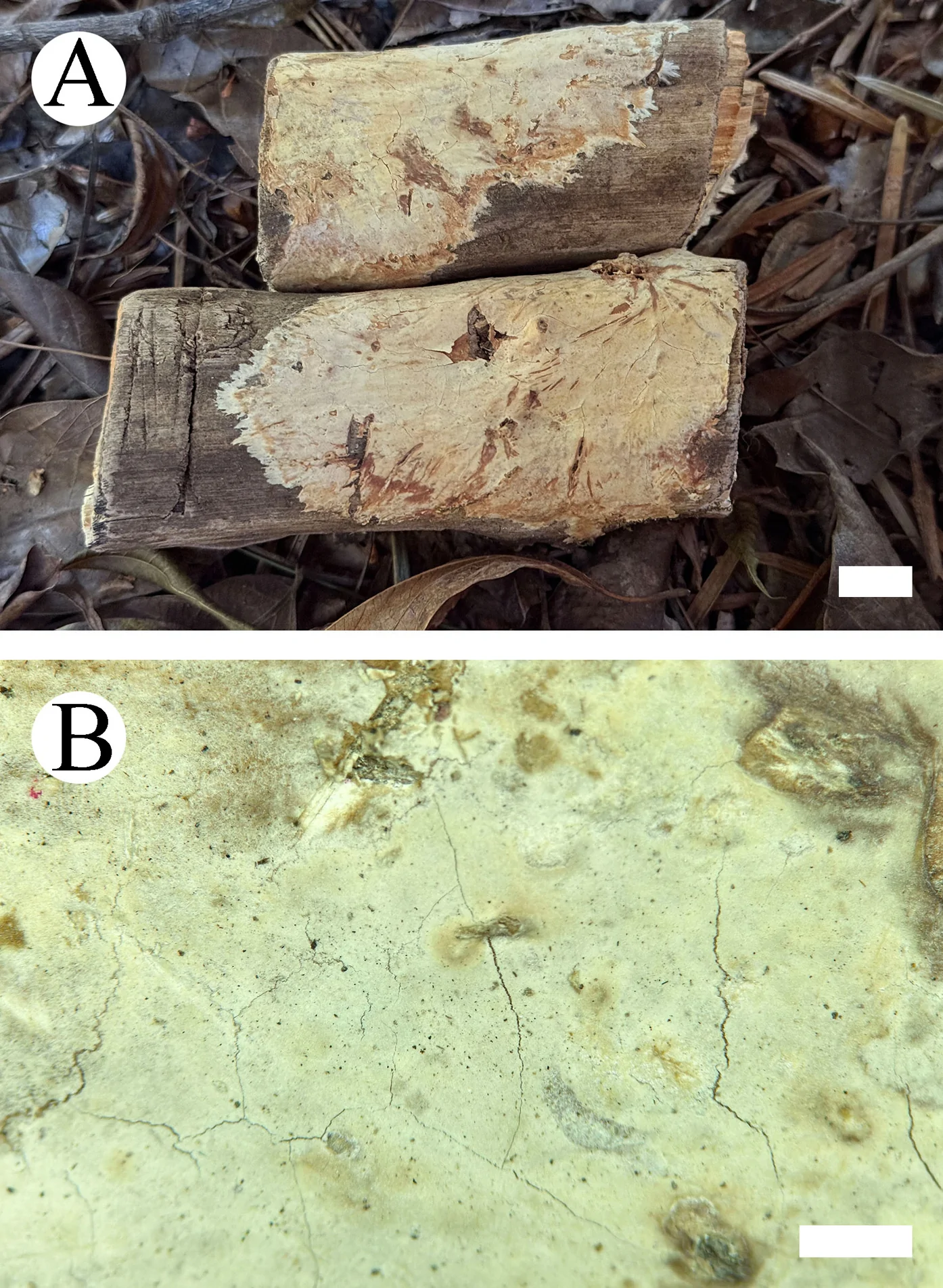
Basidiomata of *Rhizochaete membranacea* (SWFC 00020593, holotype). Scale bars: 1 cm (**A**); 2 mm (**B**).

**Figure 11.**
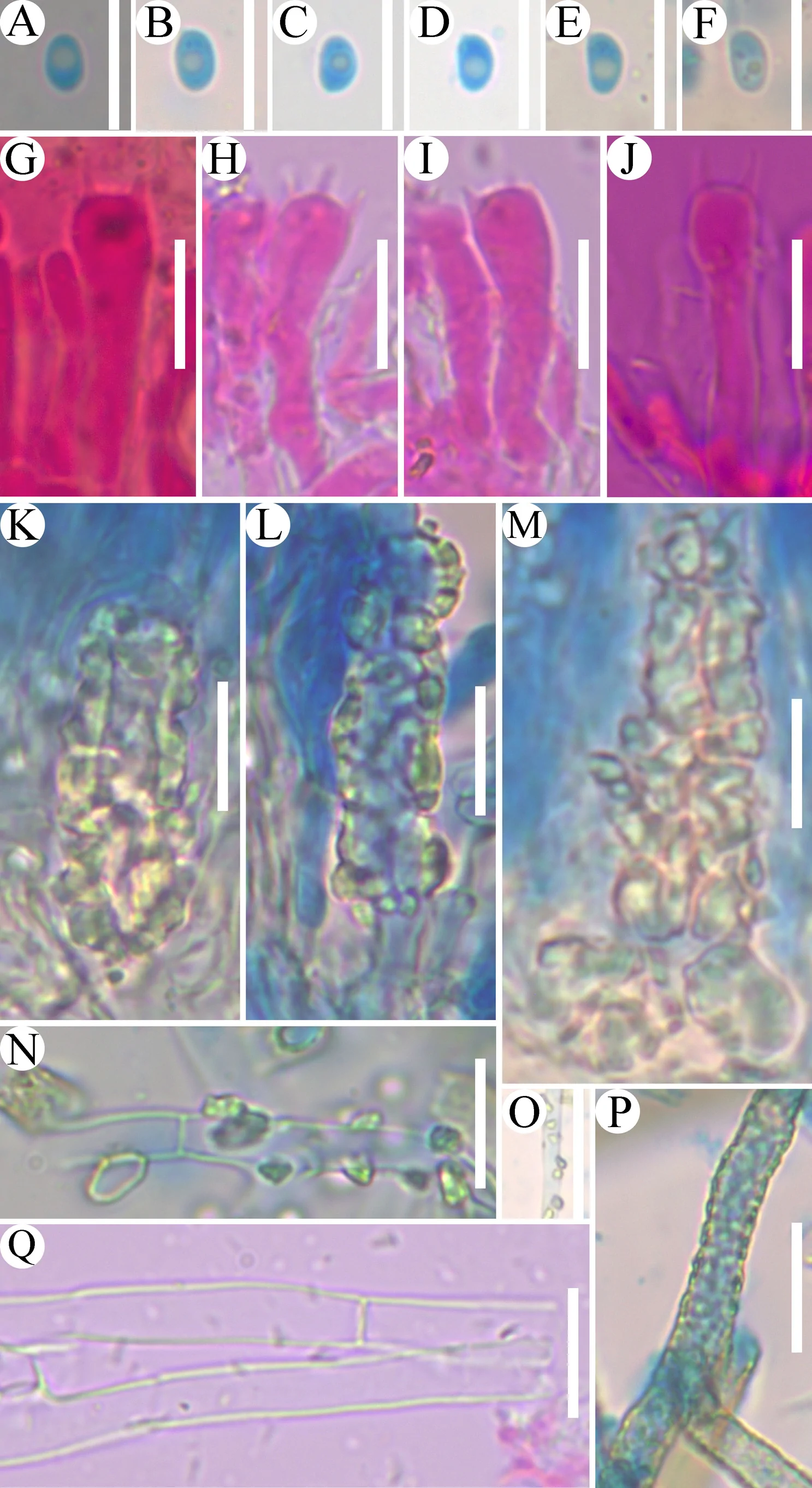
Sections of the hymenium of *Rhizochaete membranacea* (SWFC 00020593, holotype). **A–F**. Basidiospores; **G–J**. Basidia; **K–M**. Lamprocystidia; **N–Q**. Generative hyphae. Scale bars: 10 µm (**A–Q**); 10 × 100 oil.

**Figure 12.**
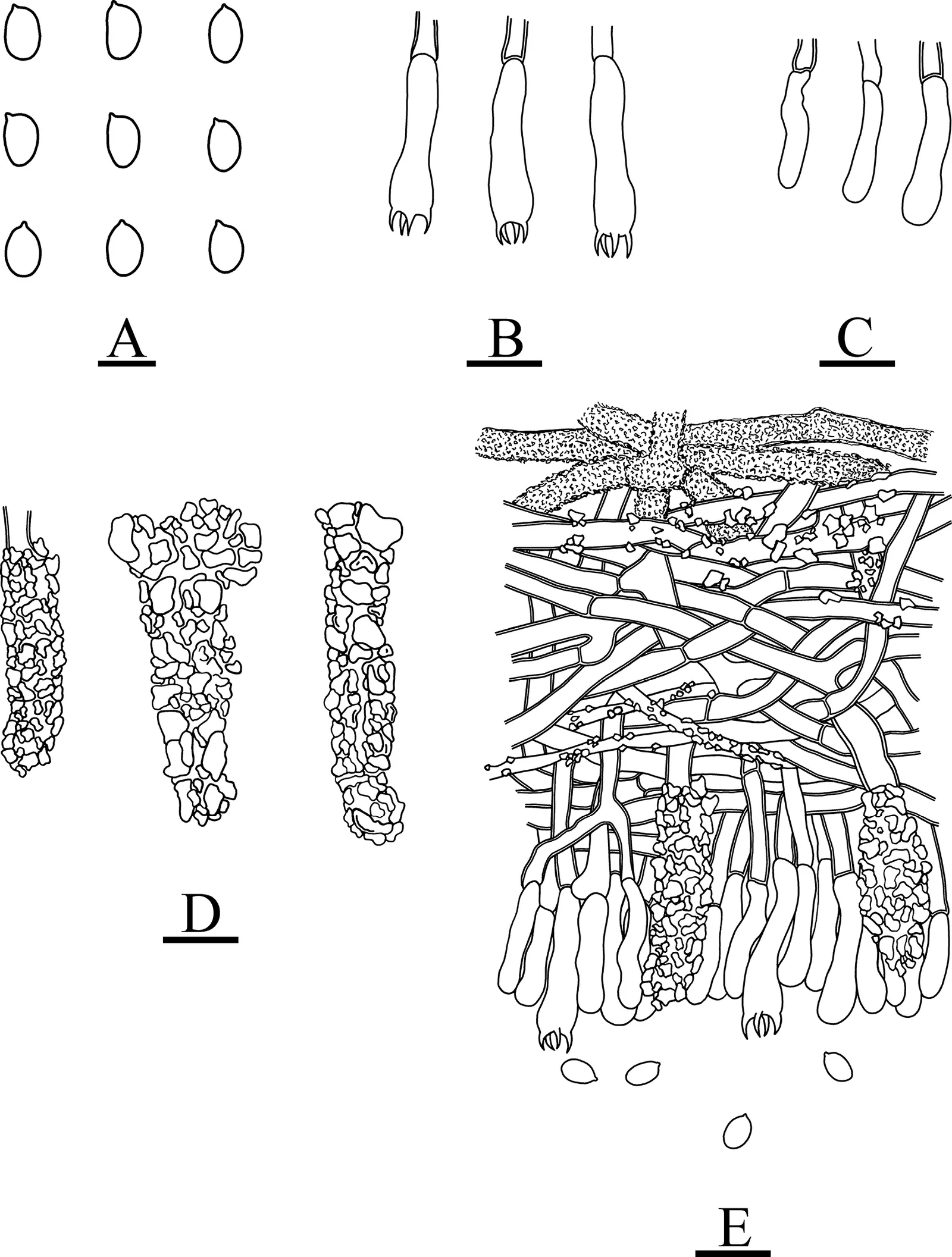
Microscopic structures of *Rhizochaete membranacea* (SWFC 00020593, holotype). **A**. Basidiospores; **B**. Basidia; **C**. Basidioles; **D**. Lamprocystidia; **E**. Part of a vertical section of the hymenium. Scale bars: 5 μm (**A**); 10 μm (**B–E**).

##### Etymology.

*Membranacea* (Lat.) refers to the membranaceous basidiomata of the type specimen.

##### Basidiomata.

Annual, resupinate, closely adnate, membranaceous, without odor or taste when fresh, becoming fragile upon drying, up to 10 cm long, 2 cm wide, 100 µm thick. Hymenial surface smooth, cracked, slightly buff when fresh, slightly buff to slightly brown upon drying. Sterile margin slightly buff, radial, up to 4 mm wide.

##### Hyphal structure.

Monomitic, generative hyphae with simple septate, colorless, thin- to slightly thick-walled, branched, interwoven, 1.5–6.5 µm in diameter; subicular hyphae encrusted with granular material; and some hyphae encrusted with crystals, IKI–, CB–; tissues unchanged in KOH.

##### Hymenium.

Lamprocystidia numerous, colorless, slightly thick-walled, encrusted with heavy crystals, 24–45 × 8–11 µm. Basidia subcylindrical, slightly constricted in the median to somewhat sinuous, with four sterigmata and a basal simple septum, 18–28 × 4–6 µm; basidioles dominant, similar to basidia in shape, but slightly smaller.

##### Basidiospores.

Ellipsoid, colorless, smooth, thin-walled, IKI–, CB–, (3.8–)4–5.5(–5.6) × (2.5–)2.7–3.1(–3.4) µm, *L* = 4.67 µm, *W* = 2.90 µm, *Q* = 1.61–1.76 (*n* = 60/2).

##### Type of rot.

White rot.

##### Additional specimen

**(paratype) examined**. China, • Yunnan Province, Zhaotong, Qiaojia County, Yaoshan Town, 27°11'N, 103°07'E, elevation 3200 m asl., on fallen angiosperm branch, leg. C.L. Zhao, 2 November 2025, CLZhao 50723 (SWFC 00050723).

## Discussion

In terms of the phylogenetic placement of the three new species, all are placed within the family *Phanerochaetaceae*, which comprises a diverse group of wood-decaying fungi that play a fundamental role in lignocellulose cycling in forest ecosystems (Floudas and Hibbett 2015). The generic-level phylogeny of *Phanerochaetaceae* has become increasingly well resolved, with several new genera erected to accommodate independent lineages (Floudas and Hibbett 2015; Miettinen et al. 2016; Luo et al. 2024). In addition, the diversity of *Phanerochaetaceae* in subtropical Asia, particularly in biodiversity hotspots such as Yunnan Province, remains inadequately characterized, and phylogenetic analyses (ITS + nLSU) were conducted to circumscribe three new species of this family: *Phanerochaete albomarginata*, *P. nigella*, and *Rhizochaete membranacea*.

Regarding phylogenetic placement, both ML and BI analyses placed *P. albomarginata* and *P. nigella* firmly within the *Phanerochaete* clade, while *R. membranacea* clustered with the *Rhizochaete* lineage (Fig. 1). Specifically, *P. albomarginata* formed a well-supported sister relationship with *P. taiwaniana* Sheng H. Wu and *P. yingjiangensis* W.Y. Xiao & H.M. Zhou, whereas *P. nigella* was sister to *P. albida* Sheng H. Wu. Morphologically, however, *P. taiwaniana* differs from *P. albomarginata* by its acute and crystal-encrusted cystidia, subclavate basidia, and smaller basidiospores (6.8–7.8 × 3.7–4.3 µm; Wu 1990). *Phanerochaete yingjiangensis* differs from *P. albomarginata* by lacking cystidia and having narrower basidia (23.5–27.0 × 3.0–4.0 µm) and cylindrical, smaller basidiospores (5.0–7.0 × 3.0–4.0 µm; Wijesinghe et al. 2025). *Phanerochaete albida* differs from *P. nigella* by its membranous basidiomata, pale ivory to white hymenial surface, narrowly clavate basidia, and smaller basidiospores (3.8–4.4 × 1.8–2.3 µm; Wu 1990). Within the *Rhizochaete* clade, *R. membranacea* appears as a distinct lineage sister to *R. yunnanensis*, but *R. yunnanensis* differs from *R. membranacea* by its grayish-yellow to brownish-orange and loosely adnate basidiomata, longer lamprocystidia (40–52 × 6–10 µm), and clavate, larger basidia (33–45 × 4.5–7 µm; Li et al. 2023).

Regarding morphological distinctiveness, *Phanerochaete albomarginata* is similar to *P. canobrunnea* Sheng H. Wu, C.C. Chen & C.L. Wei, *P. cystidiata* Sheng H. Wu, C.C. Chen & C.L. Wei, and *P. punctata* Y. Xu & C.L. Zhao in sharing membranaceous basidiomata, generative hyphae with simple septa, and ellipsoid basidiospores. However, *P. canobrunnea* differs from *P. albomarginata* by having thick-walled generative hyphae, lacking cystidia, and having shorter basidia (15–25 × 5–6 μm) and smaller basidiospores (4.2–5 × 2.5–2.8 μm; Wu et al. 2018). *Phanerochaete cystidiata* differs from *P. albomarginata* by its leptocystidia, narrower basidia (20–30 × 4.5–5.5 μm), and smaller basidiospores (4.2–5 × 2.5–2.8 μm; Wu et al. 2018). *Phanerochaete punctata* is distinguished from *P. albomarginata* by having thick-walled generative hyphae, cylindrical to subfusiform cystidia, and shorter basidia (18–22 × 5–7 μm; Xu et al. 2025). *Phanerochaete nigella* is similar to *P. citrinorhizomorpha* K.Y. Luo, Yuan Yuan, Y.C. Dai & Xin Zhang, *P. subtropica* J. Yu & C.L. Zhao, and *P. subtuberculata* J. Yu & C.L. Zhao in sharing coriaceous basidiomata, generative hyphae with simple septa, and ellipsoid basidiospores. However, *P. citrinorhizomorpha* is distinguished from *P. nigella* by having subulate cystidia and smaller basidiospores (3.5–4.5 × 1.9–2.8 µm; Luo et al. 2024). *Phanerochaete subtropica* is distinguished from *P. nigella* by having thick-walled generative hyphae, fusiform cystidia, and subcylindrical basidia (Yu et al. 2023). *Phanerochaete subtuberculata* differs from *P. nigella* by having thick-walled generative hyphae, clavate cystidia, and subcylindrical basidia (Yu et al. 2023). *Rhizochaete membranacea* resembles *R. fissurata* C.L. Zhao, *R. subradicata* Yue Li & S.H. He, and *R. terrestris* Yue Li & S.H. He in sharing a smooth hymenial surface, generative hyphae with simple septa, and ellipsoid basidiospores. However, *R. fissurata* differs from *R. membranacea* by its olivaceous-buff to pale-brown basidiomata, subfusiform to conical cystidia, and cylindrical basidia (Gu and Zhao 2021). *Rhizochaete subradicata* is distinguished from *R. membranacea* by its light-orange to grayish-orange basidiomata, longer lamprocystidia (40–75 × 7–15 µm), and clavate basidia (Li et al. 2023). *Rhizochaete terrestris* differs from *R. membranacea* by having yellow to yellowish-orange basidiomata, lacking cystidia, and having subclavate basidia (Li et al. 2023).

Regarding the limitations of the present study, several unresolved questions remain. Taxonomic research on both genera, *Phanerochaete* and *Rhizochaete*, has predominantly relied on ITS and nLSU alone; future investigations should adopt a multilocus dataset integrating both ribosomal and protein-coding loci, thereby providing a more robust foundation for taxonomic and evolutionary inferences. Research on type specimens remains limited, although such research is crucial for taxonomic studies of the genera *Phanerochaete* and *Rhizochaete*. Additionally, the limited phylogenetic signal of ribosomal markers may compromise the resolution of interspecific relationships and hinder the detection of cryptic species.

Regarding the taxonomic significance of the new taxa of the genera *Phanerochaete* and *Rhizochaete*, this study enhances understanding of *Phanerochaetaceae* diversity in subtropical Asia and provides robust phylogenetic support for the generic delineations of *Phanerochaete* and *Rhizochaete*. The new taxa are wood-inhabiting fungi, which represent an ecologically significant lineage within the tree of life, exhibiting highly specialized and diverse morphological and functional traits (Zhao et al. 2015; Luo et al. 2022; Yang et al. 2024). The three novel species described herein were all collected from decaying angiosperm wood in the subtropical forests of Yunnan Province, a region renowned for its exceptional fungal diversity. Their discovery not only expands the documented species richness of *Phanerochaetaceae* in southwestern China but also underscores the need for continued systematic surveys in undersampled tropical and subtropical regions. Notably, the abundant lamprocystidia observed in *R. membranacea* may serve a defensive function against fungivorous microarthropods, a trait previously documented in other *Rhizochaete* species (Li et al. 2023). Collectively, the present work enriches the known fungal diversity of Yunnan and establishes a solid foundation for future taxonomic, ecological, and evolutionary inquiries into wood-decaying fungi in this biodiverse region. Future investigations into the decay type (white or brown rot) and enzymatic profiles of these taxa will provide deeper insight into their ecological roles in forest carbon cycling.

## Supplementary Material

XML Treatment for Phanerochaete albomarginata

XML Treatment for Phanerochaete nigella

XML Treatment for Rhizochaete membranacea

## Additional information

### Artificial Intelligence (AI) use

The authors accept full responsibility for the content of the manuscript, including the disclosure of any use of AI.

No AI tools were used in the preparation of this manuscript.

### Author ORCIDs

Q. Li https://orcid.org/0009-0007-4990-9273

S. Wang https://orcid.org/0009-0000-1977-8507

J. Feng https://orcid.org/0009-0003-9146-6921

C. Shu https://orcid.org/0009-0008-4808-4182

C. Zhao https://orcid.org/0000-0002-8668-1075

### Data availability

All of the data that support the findings of this study are available in the main text.
